# Heavy metal and metalloid accumulation in wild brown trout (*Salmo trutta* L., 1758 *complex*, Osteichthyes: Salmonidae) from a mountain stream in Sardinia by ICP-OES

**DOI:** 10.1007/s10661-021-09204-w

**Published:** 2021-06-26

**Authors:** Angioni Alberto, Corrias Francesco, Alessandro Atzei, Sabatini Andrea, Palmas Francesco, Lai Carla, Russo Mariateresa

**Affiliations:** 1 Department of Life and Environmental Science, Chemical Food Analysis Laboratory, University of Cagliari, University Campus of Monserrato S.S. 554, Sestu. S.P. Monserrato, Bivio Monserrato Sestu Km 0, 700 Monserrato, Italy; 2 Department of Life and Environmental Science, Sustainable Development and Management of Marine and Freshwater Resources, University of Cagliari, via Fiorelli 1, 09126 Cagliari, Italy; 3 Department of Agricultural Science, Mediterranean University of Reggio Calabria, Località Feo di Vito, 89122 Reggio Calabria (RC), Italy

**Keywords:** Geochemical pollution, *Salmo trutta*, Target hazard quotient, Target cancer risk

## Abstract

This paper reports heavy metal and metalloid accumulation in wild brown trout (*Salmo trutta* L., 1758 complex) raised in freshwater and uncontaminated Sardinia system (Italy). Metals are widespread pollutants of aquatic systems, and their contamination can originate from anthropogenic activities such as industrial waste, agricultural and domestic environments, and geochemical release. Fish has a relevant position within the human diet; moreover, fishes can accumulate metals, making them a valuable tool as biomarkers for risk assessment studies. The concentration of 22 metals and metalloids after chemical digestion was assessed by inductively coupled plasma-optic emission spectroscopy (ICP-OES) in both the guts and the edible part (EP, muscle + skin) of brown trout. The results, expressed as μg g^−1^, showed different levels of accumulation in the EP and guts, following the series Cu > Zn > Ba > Al > Sr > Fe > Pb and Fe > Al > Hg > As > Mn > Cu > Ba > B > Zn > Pb, respectively. PCA analysis showed a fairly good correlation between the total lipid and SAFA content and Cd, Hg, and Pb accumulation in the gut. Non-carcinogenic risk assessment, expressed as THQ (target hazard quotient), showed values far below 1 for all metals in muscles, while high As and Hg contamination of the gut draws attention to possible health risks which should be discarded from the fish before consumption. TR (target cancer risk) values showed alarmingly high values for As and Cd when the fish were consumed entirely (gut + EP), while Pb levels were far below the safety levels.

## Introduction

Heavy metals are naturally occurring element present in trace amounts that can contaminate animals, vegetables, and fish along the food chain and are a problem for human safety (Masindi & Muedi, 2018). Maximum levels of heavy metals have been set in foodstuffs (EC 1881/2006, 2020; EC 629/2008, 2020), together with the official method of analysis (EURL, 2012).

Fish and other water organisms can be affected by water pollution, mainly accumulating chemical substances that remain in the water columns for a relatively long time (Gündoğdu & Erdem, 2008). Metals can positively affect organisms; however, they can also affect fish’s biochemical functions in terms of growth, reproduction, and wellbeing (Wang et al., 2017a, b).

The concentration of heavy metals in fish has been extensively studied over the past several decades. Researchers have shown that the degree of metal accumulation in fish is dependent on the metal type, fish species, age, sex, geographical distribution, and tissue (Petrovic et al., 2013; Ptashynski et al., 2002).

Fish and seafood products are primary components of the human diet due to their nutrients, showing high levels of healthy polyunsaturated fatty acids of the ω3 series (EPA and DHA), which are associated with a reduced heart disease risk (Corrias et al., 2020a; Psota et al., 2006; Wang et al., 2017a, b), micronutrients, and high-quality protein (Garcia-Esquinas et al., 2019; Harris et al., 2008; Lee et al., 2008; Mohanty et al., 2019).

In Italy, an average fish consumption of 30.9 ± 0.6 kg/per capita was calculated in 2017 (Eumofa, 2020). Italy was the first producer of salmonids in the EU, and *Oncorhynchus mykiss* (Pastorino & Prearo, 2015) represents the first fish from aquaculture, with 35,100 tons produced in 2017. Trout farming in Sardinia is carried out by a few small family producers, for a total harvest of 1 ton per year. To date, the only small-scale fish farm (Sadali fish farm) producing trout fry was entirely devoted to the production of Mediterranean native trout for experimental reintroduction programmes (Sabatini et al., 2011, 2018).

Since the 1970s, an evident progressive reduction in native trout presence in the original areas has been observed (Cottiglia, 1968). In Sardinia, the rivers host two non-native species: rainbow trout (*Oncorhynchus mykiss*) and brown trout of North Atlantic origin (*Salmo trutta*). The introduction of brown trout carried out with stocking programmes determined a genetic admixture between native and foreign trout belonging to the *Salmo trutta complex* (Berrebi et al., 2019; Sabatini et al., 2006, 2011; Splendiani et al., 2019).

In Sardinia, trout populations are exposed to anthropogenic stressors such as habitat fragmentation, limited water resources, and habitat pollution. However, a limited salmonid population is currently concentrated in the central-eastern part of Sardinia, where medium–low temperatures, flow regimens, and null or very low anthropic pollution characterize these habitats (Palmas et al., 2017). In this context, trout species represent an appealing model to assess the biological impact of environmental and geochemical contamination in freshwater ecosystems (Bajc et al., 2005; Davidson et al., 2009; Gündoğdu & Erdem, 2008; Linde et al., 1996, 2001).

This paper reports a contamination study of a profile of 22 metals and metalloids by ICP-OES of the edible part (EP) (muscle plus skin) and guts in wild samples (*Salmo trutta* L., 1758 complex, sensu; Bernatchez et al., 1992) collected from a mounting freshwater stream in an uncontaminated site in Sardinia. Moreover, it evaluated the non-carcinogenic (THQ) and carcinogenic (TR) human health risks associated with fish consumption. Finally, analysis of total protein, lipids, and fatty acids was carried out on fish muscles and gut for compositional studies.

## Materials and methods

### Study area and sample collection

**Fig. 1:**
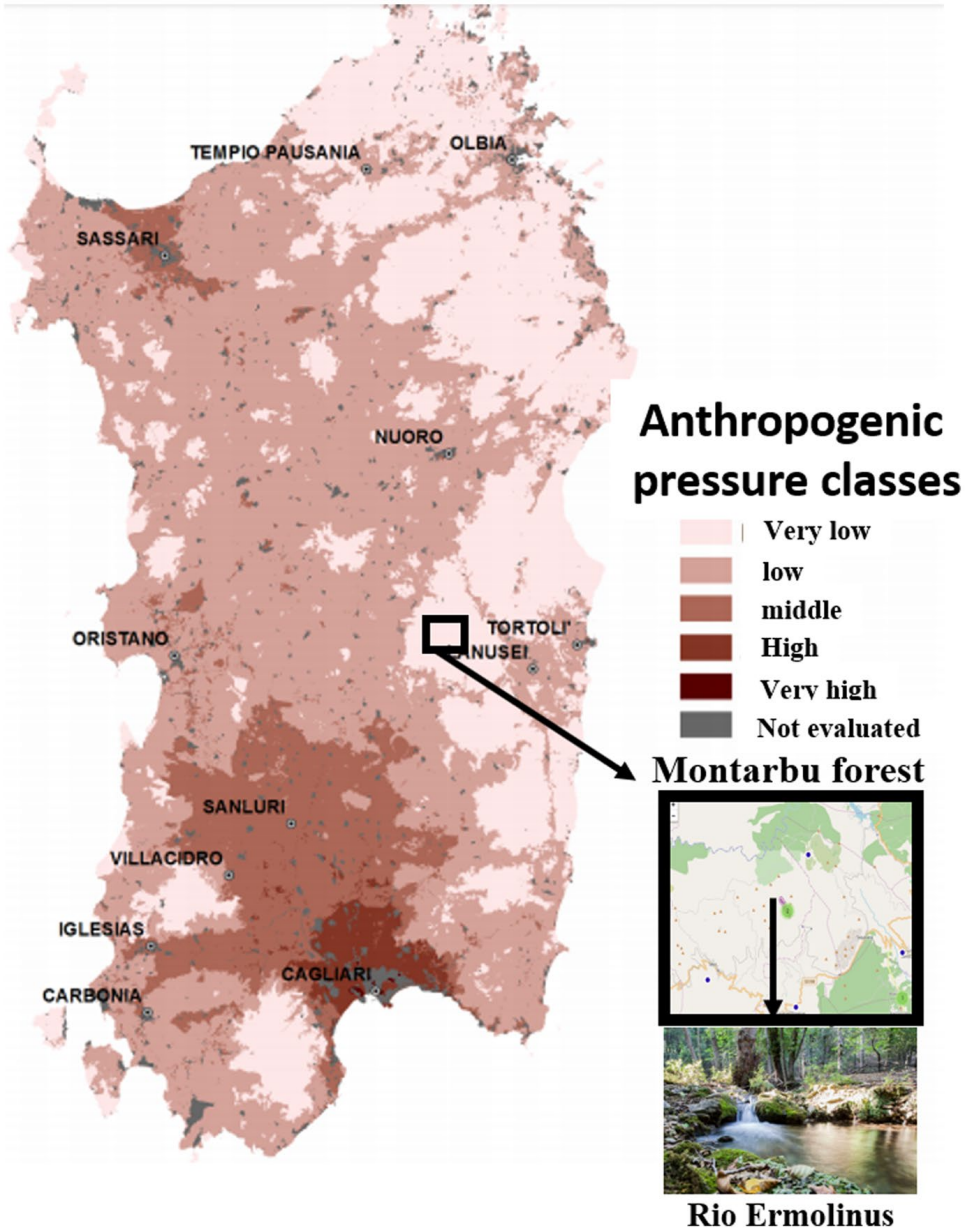
Rio Ermolinus, Montarbu Forest, and anthropogenic pressure classes in Sardina, Italy

Ten wild adult brown trout samples (> 10 cm TL) were collected in June 2018 from a headwater tributary of Flumendosa River (Ermolinus Stream, 39°52ʹN, 9°23ʹE) located in the Montarbu Forest (Sardinia, Italy) (Fig. 1). The number of fish was determined following the indications in the USEPA Guidelines (USEPA, 2000).

The stream is characterized by clear, well-oxygenated water, a moderately fast current, and the presence of waterfalls jumps, riffles runs, and pools. Moreover, the stream bed consists of sand, gravel, stones, and carbonate rocks. The carbonated part of the stream is characterized by travertine deposition (calcium carbonate) that occurs in various forms, cementing substrate particles in small dams. The abiotic parameters, water temperature, pH, water dissolved oxygen concentration, and water conductivity were recorded using a multi-parameter probe (In Situ Inc. Smart Troll MP). Trout fish were captured using low-frequency, pulsed DC electrofishing. Stunned fishes were immediately killed by spiking the brain (WOAH, 2019), held in iceboxes and transported to the laboratory. Finally, samples were frozen until analysis.

### Sample preparation

Brown trout were measured wet for total length (TL, cm), from the snout tip to the fork of the tail to the nearest 0.1 mm, using a manual calliper, and body weight (BW, g) was also determined. Then, the fish were dissected using a sharp stainless-steel scalpel, and all internal organs, including the intestine, stomach, liver, heart, kidneys, and swim bladder, were collected, weighed, and homogenized in a stainless-steel blender. The rest of the fish was separated from the head and homogenized in a stainless-steel blender. Before analysis, fish were stored in the refrigerator at 5 °C to avoid deterioration. Samples were then subjected to preparation for the analysis.

### Chemicals

Hexane, ethyl ether, petroleum ether, and methanol were of analytical grade (Sigma Aldrich Chemie, Germany). HNO_3_ 67-(69%), 30% H_2_O_2_ solution, and standards of Al, AS, B, Ba, Be, Cd, Co, Cr, Cu, Fe, Hg, Mn, Mo, Ni, Pb, Sb, Sn, Sr, Te, Ti, V, and Zn were of ICP grade (Carlo Erba Reagents Milan, Italy). HCl (34–37%) of super-pure quality (Romil Spa Cambridge, England), H_2_SO_4_ (95–98%), anhydrous Na_2_SO_4_, CuSO_4_5H_2_O, NaOH (32%), H_2_SO_4_ 0.5 N, NaOH 0.5 N, methyl red, NaCl, MgSO_4_, and KOH were of analytical grade (Sigma Aldrich Chemie, Germany). A marine oil FAME mix analytical standard was purchased from Restek (Bellefonte, PA). Double-deionized water was obtained with a Milli-Q water purification system (Millipore, Bedford, MA, USA).

### Moisture and ash

Ten grams of homogenized EP and 5 g of gut sample were weighed in a porcelain crucible and dried at 100 °C in a thermostatic heater (Argolab, Milan, Italy) for 24 h to reach a constant weight, and analyzed for moisture content assessment. Samples were then heated to 500 °C for 5 h for carbonization and ash analysis. The moisture and ash contents were calculated as a percentage of the fresh weight (FW).

### Total protein

According to the Kjeldahl digestion method, total protein was analyzed by using a BUCHI K-424 digestion unit and a BUCHI K314 distillation unit. Two grams of fresh homogenate EP and gut were weighed in a Speed-Digester flask and processed according to the method. Twenty millilitres of H_2_SO_4_ concentrate, 0.5 g of sodium sulfate, and a tip of copper sulfate were added to the mineralization flask. After mineralization, the solution was left to cool, and 50 mL of Milli-Q H_2_O was added. When the solution reached a light blue colour, the flask was inserted into the distillation unit, and a concentrated solution of NaOH was added directly to the distiller. The distillation process started when the sample solution reached a brownish-black colour. Then, 100–150 mL of distillate was collected, and a known quantity of 0.5 N plus a few drops of methyl red were added. After distillation, quantitative analysis was performed by acid–base titration using 0.5 N NaOH.

% protein = ((a-b) * c * 100 * K)/g sample.

a: mL of 0.5 N H_2_SO_4_ added to the collection flask.

b: mL of titrant used (10 mL of NaOH 0.5 N).

c: conversion factor mL of H_2_SO_4_ 0.5 N in g of nitrogen (0.007).

K: general nitrogen-protein conversion factor (6.25).

### Total lipid

One gram of fresh homogenized EP and gut were accurately weighed and extracted using the rapid extraction system for solid–liquid extraction Soxtherm (C-Gerhardt, Analytical Systems, Konigswinter, Germany) with a 150-mL mixture of ethylic ether/petroleum ether (1/1). The fat content was determined gravimetrically by weighing the boiling flask after evaporation from the extracting solvents.

### Fatty acid analysis

Fatty acid analysis was carried out according to Angioni and Addis (2014). Briefly: 1 g of EP and gut were weighed in a 15-mL screw-capped falcon tube, and then 2 mL of hexane, 1 g of NaCl, and 0.5 g of MgSO_4_ were added. The falcon tube was shaken for 3 min in a vortex, shaken for 15 min with a rotary shaker, and then centrifuged at 4000 rpm for 10 min (10 °C temperature). Transesterification was carried out as follows: 500 µL of hexane extract and 200 µL of alcoholic potash (KOH 2 N in MeOH) were heated and agitated in a vortex for 4 min. The organic phase was injected into the GC–MS instrument for analysis. A TRACE GC ULTRA Single Quad DSQ mass detector (Thermo Finnigan, Milan, Italy) equipped with a COMBI PAL autosampler (CTC Analytics, Zwingen, Switzerland), and a split/splitless injector was used. The analytical column was a Varian Factor Four VFWAX column (60 m × 0.25 mm i.d. × 0.25-µm film thickness) (Varian, Milan, Italy); helium was the carrier gas at 1 mL/min. The sample (1 µL) was injected in splitless mode (1 min). The mass spectrometer was operated with electron impact (EI) ionization and positive mode, with a solvent delay of 5.5 min. The injector, ion source, and transfer line temperatures were 50, 200, and 250 °C, respectively. Peak identification was performed comparing full mass spectra (50–550 m/z) and retention times (r.t.) from authentic standards and NIST MS Spectra Library (NIST/EPA/NIH Mass Spectral Library, 2017, Ver. 2.3).

### Heavy metal and metalloid analysis

Approximately 0.2 g of ash was accurately weighed and digested with 1.25 mL of 67% HNO_3_, 3.75 mL of 34% HCl and 1 mL of 30% H_2_O_2_ in closed polytetrafluoroethylene (PTFE) tubes using a CEM MARS 6 microwave digestion system (CEM SRL, Italy). A one-stage protocol was used as follows: heating time, 13 min; pressure, 100 PSI; and power, 600 W. After digestion, the solution was left to cool at room temperature, placed in a 10-mL flask, diluted to the mark with double-deionized water, and filtered through a 0.45-μm nitrocellulose membrane filter. Control solvent samples were simultaneously prepared to avoid false positives and contamination during analysis. Hg and As sample preparations were carried out using an Agilent VGA-77 instrument (Agilent, Milan, Italy) according to the manufacturer instructions (Beach, 2010; Evans et al., 2010). Analysis was carried out using a Varian 710ES ICP optical emission spectrometer (Agilent, Milan, Italy), according to Corrias et al. (2020b). Each measurement was conducted in triplicate. The limits of detection (LODs) and the limits of quantitation (LOQs) were calculated as three and ten times the standard deviation reading of the blank sample signal, respectively. Calibration curves were calculated with five points starting from the LOQ value and were considered acceptable when r^2^ ≥ 0.995.

### Risk assessment

#### Non-carcinogenic risk evaluation

The target hazard quotients (THQ) index was applied to assess the potential non-cancer risk associated with consumption of the trout sampled and calculated following the USEPA guidelines (1989) (1):

1 $$\mathrm{T}\mathrm{H}\mathrm{Q}=\mathrm{E}\mathrm{F}\mathrm{x}\mathrm{E}\mathrm{D}\mathrm{x}\mathrm{I}\mathrm{R}\mathrm{x}\mathrm{C}/{\mathrm{R}\mathrm{f}\mathrm{D}\mathrm{x}\mathrm{B}\mathrm{W}\mathrm{x}\mathrm{A}\mathrm{T}}_{n}$$

The average body weight for an adult consumer of 67 kg and an average fish intake rate (IR) of 0.036 kg/day for a person (FAO, 2020) were considered for the THQ calculation (SI trout Table A). The reference doses (RfDs) for oral intake were obtained from the (Integrated Risk Information System 2020; USEPA, 2010). The THQ values were calculated for the traditional edible part and the sum of EP and the gut. THQ values exceeding “1” indicate a potential health risk to consumers (USEPA, 1989). Regarding Hg, we considered the RfD of methyl mercury, assuming that all mercury found was in this organic form (MeHg).

#### Carcinogenic risk evaluation

As Cd and Pb are known to pose a risk of cancer (IARC, 2012). IARC has classified MeHg as “possibly carcinogenic to humans” (IARC, 2012), whereas the US Environmental Protection Agency (USEPA) has established that evidence of MeHg carcinogenicity in humans is insufficient. Indeed, the rationale of the carcinogenicity in experimental animals was restricted; therefore, the USEPA has designated MeHg as a Group C material (possible human carcinogen). Since the CPSo (Carcinogenic Potency Slope oral) of Cr and Hg has not been published by the USEPA, TR (Target Cancer Risk) was calculated only for As (CPSo = 1.5 mg/kg/day), Cd (CPSo = 6.3 mg/kg/day), and Pb (CPSo = 8.5 × 10^−3^ mg/kg/day) (USEPA, 2010). The risk of cancer was estimated as the probability of an individual developing cancer over a lifetime as a result of exposure to potential carcinogens using the target cancer risk (TR) (2):

2 $$\mathrm{T}\mathrm{R}={\mathrm{E}\mathrm{F}\mathrm{x}\mathrm{E}\mathrm{D}\mathrm{x}\mathrm{I}\mathrm{R}\mathrm{x}\mathrm{C}\mathrm{P}}_{\mathrm{o}}\mathrm{x}\mathrm{C}/{\mathrm{B}\mathrm{W}\mathrm{x}\mathrm{A}\mathrm{T}}_{\mathrm{c}}$$

The exposure duration (average lifetime) was set at 83 years, the average life expectancy (both sexes) in Italy. Acceptable risk levels for carcinogens have been set in the range from 10^−4^ to 10^−6^.

### Statistical analysis

Analysis of variance (ANOVA) was carried out with XLSTAT software (Addinsolf LTD, Version 19.4). Mean comparisons of the effects of treatments were calculated by Fisher’s least significant difference test at p ≤ 0.05. ICP-OES, GC/MS fatty acid, and lipid analysis data were analyzed with SIMCA 14 (Umetrics AB, Umea, Sweden) for principal component analysis (PCA). The coefficient of determination (R2) was considered to evaluate the correlation between the lipid fraction and metals accumulation (Atherton et al., 2006).

## Results

Samples of brown trout ranged from 83.52 to 140.48 g in body weight (BW) and from 15.19 to 25.53 cm in total length (TL), while the gut weight ranged from approximately 10.56 ± 8.91 g (± RSD). The EP’s water and ash contents were 74.83 ± 1.38 and 2.66 ± 11.21 g/100 g FW, respectively, which were slightly higher than the gut content (Table 1). Protein and lipids accounted in for 17.84 ± 3.19 g/100 g FW and 4.67 ± 19.26 g/100 g FW (g ± RSD%), respectively (Table 1), whereas in the gut were 11.51 ± 8.09, and 2.23 ± 19.10 (g ± RSD%). SAFAs (36.64 ± 4.35%) and MUFAs (36.00 ± 4.41%) were the most abundant fatty acids, followed by PUFAs (27.34 ± 4.20%) (Table 2). Gut fatty acid followed PUFA > MUFA > SAFA, accounting for 37.74 ± 8.78%, 23.92 ± 13.35% and 14.67 ± 8.81%, respectively (Table 2).

**Table 1:** Chemical-physical parameters of the fish used in the experiment

	Average weight		Humidity	Ash	Total protein	Total lipid
	g ± RSD%	% whole fish	g/100 g FW*	g/100 g FW	g/100 g FW	g/100 g FW
Muscles	93.51 ± 17.41	89.44 ± 1.05	74.83 ± 1.38	2.66 ± 11.21	17.84 ± 3.19	4.67 ± 19.26
Gut	11.02 ± 18.79	10.56 ± 8.91	70.07 ± 2.29	2.10 ± 8.15	11.51 ± 8.09	2.23 ± 19.10

^*^g/100 g ± RSD%

**Table 2:** Fatty acid composition (%) of trout muscles collected from Sardinia mounting streams

	Muscles	Gut
Σ saturated	36.64 ± 4.35	14.67 ± 8.81
Σ Monounsaturated	36.00 ± 4.41	23.92 ± 13.35
Σ polyunsaturated	27.34 ± 4.20	37.74 ± 8.78
Σ FFA	0.026 ± 6.54	-
PUFA/SAFA	0.75 ± 14.48	2.59 ± 12.11
Σɷ3 (%)	8.84 ± 7.07	21.13 ± 11.80
Σɷ 6 (%)	15.75 ± 6.40	16.61 ± 10.12
ɷ3/ɷ6	0.56 ± 6.15	1.28 ± 12.22

The C16 and C18 families were the most represented among SAFAs, MUFAs, and PUFAs. C18:3α (4.79 ± 10.55%), EPA (2.56 ± 11.29%), and DHA (1.14 ± 13.64) were also present in considerable amounts (Table 3); ω3 families accounted 8.84 ± 7.07%; and ω6 fatty acids accounted for 15.75 ± 6.40%, leading to a ω3/ω6 ratio of 0.56 ± 6.15 in EP, whereas in the gut ω3 accounted 21.13 ± 11.80% and ω6 16.61 ± 10.12%, with a ratio ω3/ω6 1.28 ± 12.22% (Table 2).

**Table 3:** Fatty acid composition (%) of the lipid fraction extracted from the muscles of the brown trout

Fatty acid	Trout muscles
C14:0	3.36 ± 13.76
anteiso C15:0	0.44 ± 14.18
C15:0 + isomer	0.39 ± 16.79
anteiso C16	0.13 ± 16.46
C16:0	25.61 ± 4.92
C17:0	0.10 ± 17.07
C17:0 anteiso C18	0.47 ± 13.13
C18:0	5.29 ± 8.18
C20:0	0.17 ± 9.80
C14:1	0.21 ± 12.25
C16:1	14.31 ± 12.82
C16:1 n9 7 methyl	0.49 ± 11.95
C17:1	0.50 ± 17.25
C18:1c	16.52 ± 13.31
C18:1 Δ11	3.60 ± 9.47
C20:1	0.36 ± 17.13
C16:2	1.20 ± 7.54
C18:2	14.28 ± 6.61
C18:2n6c	0.33 ± 12.65
C18:3	0.24 ± 39.66
C18:3α	4.79 ± 10.55
C18:4n3	0.62 ± 12.31
C20:2	0.45 ± 12.08
C20:3	0.27 ± 13.76
C20:4	0.70 ± 15.43
C20:3n3 (11–14-17)	0.13 ± 15.14
C20:4n3	0.22 ± 16.51
C20:5 EPA	2.56 ± 11.29
C22:5	0.42 ± 13.74
C22:6 DHA	1.14 ± 13.64
MonoM (9,5) FA	0.009 ± 12.75
Dime (9,5) FA	0.001 ± 23.87
DiMe (11,3) FA	0.011 ± 14.38
MonoM (11,5) FA	0.0004 ± 14.93
Dime (11,5) FA	0.005 ± 9.57

The ICP-OES method allowed the detection and quantitation of 22 metals, with calibration curves with correlation coefficients ranging from 0.9922 to 0.9999, and LOQ values suitable for the analysis and in line with literature data (Wenzl et al., 2016) (Table 4). Be, Co, Mo, Sb, Sn, and Te were not detected in any samples. The detected metals and metalloids showed high variability among the different fishes. Fe, Al, Hg, As, and Mn showed relatively high gut concentrations, with residue values of 226.98 ± 39.18, 80.69 ± 41.90, 12.23 ± 16.16, 10.57 ± 44.03, and 8.39 ± 32.67 μg· g^−1^ (± RSD%), respectively. In contrast, the other metals and metalloids showed values in the range of 0.03 ± 25.02 (Cd) and 4.89 ± 65.56 μg· g^−1^ (Cu) (Table 4). The EP showed general values always below the gut, with the most abundant metals being Cu (1.19 ± 60.87 μg g^−1^), Zn (0.97 ± 15.96 μg g^−1^), Ba (0.80 ± 38.48 μg g^−1^), Al (0.70 ± 55.10 μg g^−1^), and Sr (0.67 ± 16.70 μg g^−1^). Moreover, As, B, Ni, and Ti were absent in the edible part (Table 4).

**Table 4:** Concentration of metals and metalloids (μg g^−1^, FW) in guts and muscle of brown trout

Metal	λ	MRL, μg g^−1^	LOQ, μg g^−1^	Linear regression equation	R^2^	Gut, μg g^−1^ ± RSD	Muscle, μg g^−1^ ± RSD
Al	237.3		0.10	y = 717.36x + 12.11	0.9990	80.69 ± 41.90	0.70 ± 55.10
AS	188.98		0.10	y = 79.21x − 11.44	0.9996	10.57 ± 44.03	< LOQ
B	249.77		0.025	y = 5251x + 789.8	0.9997	2.80 ± 36.32	< LOQ
Ba	493.40		0.005	y = 8019–417.3	0.9995	4.22 ± 16.70	0.80 ± 38.48
Be	313.04		0.005	y = 114,687x − 10,873	0.9989	< LOQ	< LOQ
Cd	226.50	0.05	0.005	y = 15,189x + 3513	0.9922	0.03 ± 25.02	0.03 ± 18.65
Co	228.61		0.025	y = 2213x − 3.07	0.9998	< LOQ	< LOQ
Cr	267.71		0.005	y = 10,136x − 23	0.9999	0.12 ± 27.14	0.08 ± 23.95
Cu	324.75		0.01	y = 17,524x − 363	0.9997	4.89 ± 65.56	1.19 ± 60.87
Fe	259.94		0.01	y = 3069x + 150	0.9995	226.98 ± 39.18	0.30 ± 34.79
Hg	194.16	0.50	0.05	y = 397x + 47	0.9998	12.23 ± 16.16	0.08 ± 14.89
Mn	257.61		0.005	y = 108,182x − 588	0.9996	8.39 ± 32.67	0.04 ± 22.33
Mo	204.59		0.10	y = 450x − 46	0.9959	< LOQ	< LOQ
Ni	216.55		0.025	y = 1699x − 19	0.9997	0.11 ± 44.40	< LOQ
Pb	220.35	0.3	0.05	y = 329x + 16	0.9989	0.69 ± 48.42	0.20 ± 48.85
Sb	217.58		0.50	y = 46x − 10	0.9981	< LOQ	< LOQ
Sn	189.92		0.50	y = 79x + 1	0.9990	< LOQ	< LOQ
Sr	407.77		0.005	y = 827,346x + 17,943	0.9991	0.38 ± 15.49	0.67 ± 16.70
Te	214.28		0.50	y = 116x + 7	0.9974	< LOQ	< LOQ
Ti	336.12		0.10	y = 14,629x + 1965	0.9989	1.12 ± 19.96	< LOQ
V	292.40		0.005	y = 14,788x − 37	0.9998	0.14 ± 27.77	0.01 ± 26.34
Zn	213.85		0.025	y = 5027x − 50	0.9991	0.78 ± 25.19	0.97 ± 15.96

Non-carcinogenic risk assessment calculated considering a regular intake of approximately 0.036 kg/day fish resulted in THQ values below 1 for each metal in the EP, with maximum and minimum values ranging from 4.30 × 10^−1^ (Hg) and 2.87 × 10^−5^ (Cr), respectively (Table 5). Also, the intake of EP plus the gut showed the minimum THQ values for Cr (7.16 × 10^−5^) and maximum values for As and Hg of 18.93 and 66.14, respectively (Table 5).

**Table 5:** Target hazard quotients (THQ) and target cancer risk (TR) caused by consuming brown trout muscles and gut

Metal	RfD, mg/kg/day	THQ muscles	THQ gut + muscle	CPS, mg/kg/day	TR muscles	TR gut + muscle
Al	1.0	3.76 × 10^−04^	4.37 × 10^−02^	-	-	-
AS	3.0 × 10^−04^	-	18.93	1.5	-	8.52 × 10^−3^
B	2.0 × 10^−01^	-	7.52 × 10^−03^	-	-	-
Ba	2.0 × 10^−01^	2.15 × 10^−03^	1.35 × 10^−02^	-	-	-
Cd	1.0 × 10^−03^	1.61 × 10^−02^	3.22 × 10^−02^	6.3	1.02 × 10^−4^	2.03 × 10^−4^
Cr	1.5	2.87 × 10^−05^	7.16 × 10^−05^	-	-	-
Cu	4.0 × 10^−02^	1.60 × 10^−02^	8.17 × 10^−02^	-	-	-
Fe	7.0 × 10^−01^	2.30 × 10^−04^	1.74 × 10^−01^	-	-	-
Hg	1.0 × 10^−04^	4.30 × 10^−01^	66.14	-	-	-
Mn	1.4 × 10^−01^	1.54 × 10^−04^	3.24 × 10^−02^	-	-	-
Ni	2.0 × 10^−02^	-	2.96 × 10^−03^	-	-	-
Pb	3.5 × 10^−03^	3.07 × 10^−02^	1.37 × 10^−01^	8.5 × 10^−03^	9.13 × 10^−7^	4.06 × 10^−6^
Sr	6.0 × 10^−01^	6.00 × 10^−04^	9.40 × 10^−04^	-	-	-
Ti	-	-	-	-	-	-
V	9.0 × 10^−03^	5.97 × 10^−04^	8.96 × 10^−03^	-	-	-
Zn	3.0 × 10^−01^	1.74 × 10^−03^	3.13 × 10^−03^	-	-	-

Carcinogenic risk expressed as TR showed values for Cd and Pb of 1.02 × 10^−4^ and 9.13 × 10^−7^ in the EP and 2.03 × 10^−4^ and 4.06 × 10^−6^ in the EP + gut. As residues in the EP were below the LOQ of the analytical method, the TR calculated for the total fish was ascribable only to the gut's contamination (8.52 × 10^–3^).

**Fig. 2:**
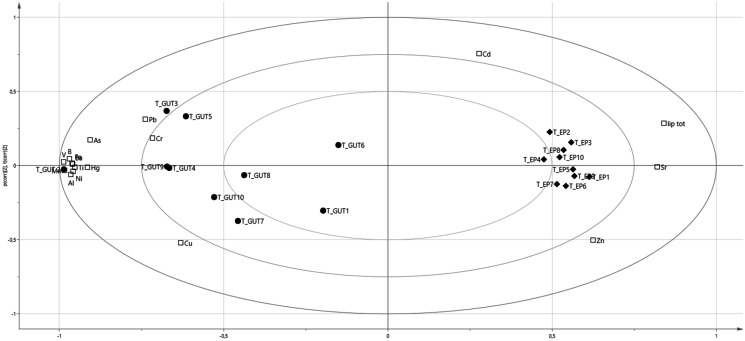
The analysis of PCA biplot score and loadings

**Fig. 3:**
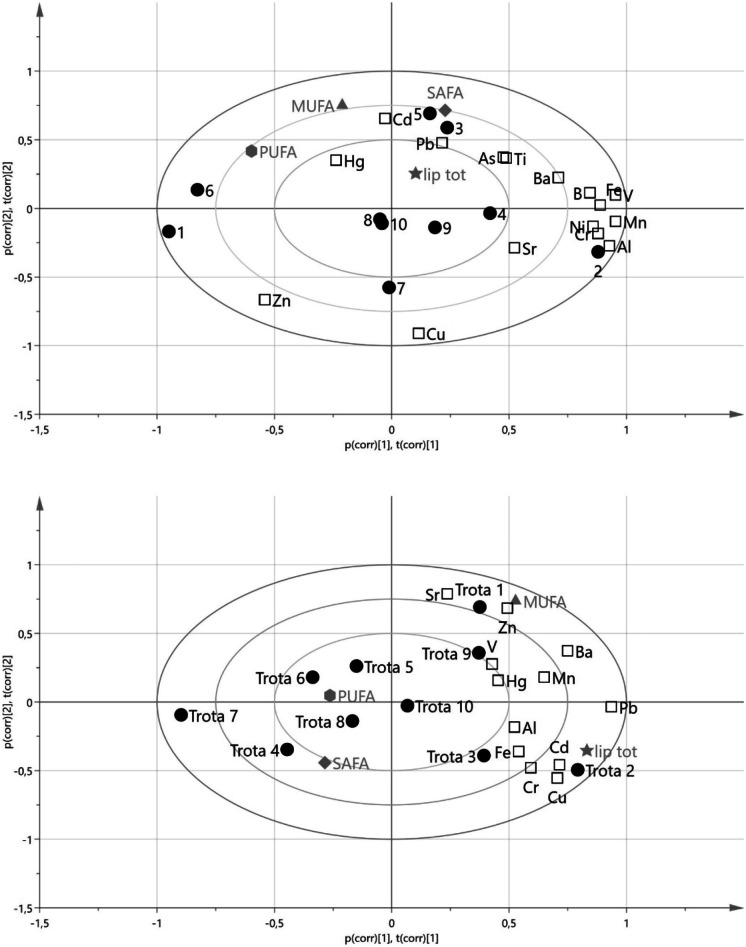
The PCA biplot loadings. **a** Correlation of PUFAs to heavy metal accumulation in the gut. **b** Influence of SAFA and PUFA in metal accumulation

Principal component analysis (PCA) was used to verify the correlation between metals and metalloid accumulation and the lipidic or fatty acid amount in the gut and EP. The analysis of PCA biplot score and loadings for gut showed that the samples of trout had an uneven composition (Fig. 2); however, the lipid fraction showed a fairly good correlation with Cd (0.7207) and Hg (0.7557). The PCA biplot loadings showed for total lipids a significant influence along the PC2 axis of the variable Hg, Cd, and Pb, whereas Cd and Pb contribute for SAFA and Cd and Hg for MUFA; PUFAs were not correlated to heavy metal accumulation in the gut (Fig. 3a).

Correlation analysis for EP showed a fairly good correlation among total lipids (between 0.539 and 0.7877), Cd, Pb, Cu, Fe, and Cr, whereas MUFA showed a low correlation with Cd (0.4737) and Hg (0.4312), PUFA a negative correlation with Al (− 0.6481) and Cd (− 0.4805), and SAFA had no correlation with metals accumulation. The most influencing variables for total lipids loadings were Cd, Pb, and Cu along the PC1 axis and MUFA Cd, Hg, and Zn. SAFA and PUFA fell in the PC1 negative loadings and did not influence metals accumulation (Fig. 3b).

PCA emphasizes the differences in accumulation between the two matrices; Pb and Hg were the most discriminating along the PC1 axis, while Cd was the most discriminating on the PC2 axis (Fig. 2).

## Discussion

Wild brown trout in nature usually feed on invertebrates belonging to terrestrial and aquatic communities (Fochetti et al., 2003). Feeding patterns affect both lipid and protein profiles in a relevant way (Oz, 2016). Therefore, the profile of fatty acids can change drastically, and the degree of correlation with the diet is not always unambiguous (Oz, 2019; Aziz et al., 2013; Trbovic et al., 2012; Bell & Dick, 2004). Sardinian brown trout mainly feed on aquatic insects (Massidda et al., 2008), which present fatty acid compositions with comparable amounts of SAFAs and MUFAs and low levels of PUFAs (Fontaneto et al., 2011; Kiyashko et al., 2004; Shipley et al., 2012), reflecting the composition found in this paper.

The bioaccumulation of persistent contaminants in living organisms depends on on-site pollution, detoxification rate, and metabolism (Jakimska et al., 2011). This phenomenon directly affects both fish tissues and organs, leading to increased fish mortality due to exceeding acute limits (Davidson et al., 2009; Pasha, 2016). Also, a transfer of contaminants to humans through the food chain may occur. Biomonitoring studies represent a necessary tool to evaluate the environmental behaviour of elements potentially harmful for both humans and other living organisms (Corrias et al., 2020b). Anthropogenic activities are considered the primary source of metal pollution in aquatic ecosystems. Thus, several studies in the literature deal with human activities’ effect on environmental wellbeing, especially concerning heavy metal pollution from industrial waste (Gaur et al., 2020; Masindi & Muedi, 2018).

Data on the bioaccumulation of metals and metalloids in aquatic organisms related to the surrounding environment’s natural and geological characteristics are still lacking. However, some data showed an increased concentration of metals in volcanic soils and surrounding groundwater (Andronico et al., 2009; Buat-Menard & Arnold, 1978; Favalli et al., 2004). Cyclic siliciclastic depositions characterize the Montarbu area in the water basin. Stratigraphy showed episodic deposition of carbonate beds and volcanic products (Costamagna, 2019). Mica from the phyllosilicate family formed by parallel sheets of silicate that characterize the Ermolinus River shows residues of Al, K, Mn, Fe, Zn and Ca (Charette & Sholkovitz, 2006). Moreover, volcanic terrains can show high Ba, As, and Hg levels from the Earth’s crust and poured onto the ground over centuries through volcanic eruptions (WHO, 2011).

The Ermolinus River is in the inner part of the Montarbu forest, which is an area with shallow anthropogenic class pressure (Fig. 1), is hard to reach, and is not subjected to wind coming from polluted areas. The chemical-physical characteristics of the Ermolinus stream showed ideal values for life in freshwater streams, indicating a healthy ecosystem. With a temperature of 15.2 °C in the May/June period, Ermolinus river water showed good resilience to the atmospheric temperature (20 °C) (SI Trout Table B). The pH at 7.5 was in the middle of the range for potable freshwater established by the OMS (6.5–8.5) and was weakly basic (WHO, 2007). The pH value influences metals’ solubility; Fe and Cu are more soluble at pH values < 7, and Al and Zn are more soluble at pH values > 10. Moreover, this value corresponds to the pH of fish blood and therefore maintains the body’s homeostasis. The dissolved oxygen content was 8.33 mg/L, within the suggested range of 7–11 mg/L (Rounds et al., 2013). Mountain rivers, belonging from rainfalls and melting snow, show conductivity values below 50 μS/cm, while levels above 500 μS/cm can be found in lowland rivers, where inorganic matter can accumulate from sediments and natural origin. The conductivity value detected in the Ermolinus River (648 µS/cm) was slightly higher than the suggested levels for freshwater streams (50–500 μS/cm) and showed that the water in the ponds belongs to springs that extract minerals from rocks, enriching the mineral content in the water (White, 2010) and probably influencing metal accumulation in trout.

As expected, the levels of the 22 metals and metalloids reported in the present study showed both a different distribution and concentration ratio among the gut and EP. Residue levels were higher in the gut than in the EP for all compounds except for Sr and Zn. Indeed, gut samples included the liver and other organs that accumulate metallothionein proteins; these proteins are involved in homeostatic regulation and the detoxification process from heavy metals and can react strongly with metals (Wang et al., 2014). Al, Mn, Fe, Cu, and Zn are essential elements because of their important role in biological systems. Mn’s absence results in severe skeletal and reproductive aberrations in mammals (Sivaperumal et al., 2007). Cu is a part of several enzymes and is necessary for haemoglobin's biosynthesis with Fe (WHO, 1989). Zn is an essential trace metal for preventing retarded growth, loss of taste and hypogonadism, and fertility decrease (Sivaperumal et al., 2007). Cr, Ni, and V are considered essential metals involved in glucose metabolism (Cr), normal growth and reproduction in animals and humans (Ni), and cell growth and essential components of some enzymes (V) (Ahmed et al., 2016). However, when consumed in high amounts, it can result in severe toxicity (Calabrese et al., 1985; Malik et al., 2010). In contrast, Sr, Ba, B, and Ti’s biological functions in organisms are still poorly understood, and they are considered non-essential metals (Carvalho et al., 2005). The most abundant metals in the EP and the gut followed the series Cu > Zn > Ba > Al > Sr > Fe > Pb and Fe > Al > Hg > As > Mn > Cu > Ba > B > Zn > Pb, respectively. Cd, Cr, Hg, Mn, and V were present only in trace amounts in the EP, whereas Cd, Cr, Ni, and V were detected in the gut.

Metals such as As, Cd, Pb, and Hg do not play any metabolic function. In contrast, these metals are considered toxic elements and harmful for humans, even at low concentrations, when ingested over a long period (Tchounwou et al., 2012). According to the European Commission, the allowed limits for Cd, Pb, and Hg in fish for human consumption are 0.05, 0.3, and 0.5 μg g^−1^, respectively (EC 1881/2006, 2020; EC 629/2008, 2020). In this study, the EP samples always showed values lower than the EU Regulation limits. In contrast, considering the gut, Hg and Pb showed values 24 times and 2.3 times higher than their MRLs, respectively.

Among the metals and metalloids found in trout samples, only thirteen were investigated by other authors. Cd, Cu, Pb, Hg, and Zn were the most studied in brown trout, while only a few papers investigated the amounts of As, Ba, Co, Cr, Fe, Mn, Ni, and V. Analysis was carried out on the muscles and the liver, showing values higher in the liver than in the muscles for most compounds (Table 6). The Cd content ranged from 0.02 to 0.04 μg g^−1^ in this study, in both the EP and gut, with average values similar to those found by Dvorak et al. (2016) in brown trout in the Czech Republic but markedly lower than those found in other surveys (Table 6). The average levels of Cu were similar to those found by Linde et al. (2004) and Monna et al. (2011) but lower than those found by Vitek et al. (2007) in muscles. Moreover, the Cu levels found in the gut were markedly lower than those detected by other authors in the liver (Table 6).

**Table 6:** Literature data on brown trout metals bioaccumulation in a free environment, and aquaculture

Fish species	Location	Wet		As	Ba	Cd	Co	Cr	Cu	Fe	Hg	Mn	Ni	Pb	V	Zn	Rerference
Brown Trout	Asturian rivers	μg g^−1^	Liver			0.165–7.957			13.78–322.85					0.88–13.63			Linde et al. (1996)
Brown Trout	Ferrerias, Raices rivers	μg g^−1^	Muscle			0.001–0.026			0.173–0.278		0.069–0.215			0.001–0.026			Linde et al. (2004)
			Liver			0.231–0.91			20.46–70.41		0.052–0.657			0.036–0.275			
Rainbow trout	Turkey, Karakaya Res	μg g^−1^	Muscle	0.052–0.103		0,00–0,0019	0.575–0.904	0.275–0.692	0.23–0.524	2.533–13.297		0.505–0.951	0.792–1.362	0.042–0.063		2.035–4.974	Varol et al. (2017)
Rainbow trout	New Zealand	μg g^−1^	Muscle	0.01–0.09							0.03–1.56						Robinson et al. (1995)
			Liver								0.01–4.15						
Salmonoids	Slovenian rivers	μg g^−1^	Muscle + skin						< 0.05–1.38	2.6–7.7		< 0.07–0.35				5.2–17.6	Bajc et al. (2005)
			Liver						3.43–324	54–501		0.40–2.61				10.6–87.2	
			Head						< 0.05–0.82	5.0–70.2		0.21–3.89				19.5–62.1	
			Kidney						0.29–4.02	61.325		0.16–1.25				14.8–46.9	
Brown Trout	Louãka River	μg g^−1^	Muscle			0.003–0.026		0.028–0.073	0.329–0.437		0.065–0.106		0.058–0.102	0.108–1.010		3.956–5.801	Vitek et al. (2007)
			Liver			0.107–0.223		0.035–0.068	59.97–145.80		0.123–0.159		0.060–0.328	0.073–1.721		30.67–34.27	
Brown Trout		μg g^−1^	Muscle			0.001–0.17			0.048–1.48					0.01–0.95		2.48–43.7	Monna et al. (2011)
			Liver			0.037–8.3			1.6–92.9					0.047–24.0		11.3–102	
Different species	Gaza	μg g^−1^	Muscle			< LOD–0.090			0.251–0.907			0.376–0.834	0.453–0.978	< LOD–0.552		3.70–20.53	Elnabris et al. (2013)
Carps	Lhasa, Tibet	μg k g^−1^	Muscle	13.69–286.51	13.87–474.0	n.d. –4.24	3.84–8.35	2.36–17.93	135.22–257.76			127.71–778.97		21.41–43.56	6.16–13.81		Jiang et al. (2014)
			Gill	35.17–218.65	764.8–13,957	5.36–10.34	37.77–124.81	41.26–107.39	455.82–840.07			4051–109,818		93.75–296.74	47.10–167.83		
			Liver	50.35–607.07	7.11–62.22	12.22––86.18	21.75–83.05	12.42–29.18	600.26–823.97			251.14–1520.75		65.03–367.47	29.99–107.07		
			Heart	35.45–412.50	11.30–227.25	7.52–70.41	12.17–546.03	n.d. –13.91	2260–3799			484.63–735.91		24.26–131.96	9.62–143.36		
Brown Trout	Czech Republic	μg g^−1^	Muscle			< 0.02–0.03					0.25–0.61			< 0.10–0.22			Dvořák et al. (2016)
Rainbow trout	Yasuj, Iran	μg g^−1^	Muscle			0.105					0.022			1.07			Majlesi et al. (2019)
Different species	India	μg g^−1^	Muscle			n.d. –26.06			0.52–10.51					1.72–22.03		9.24–81.5	Arumugam et al. (2018)
Brown Trout	Ermolinus river Sardinia, Italy	μg g^−1^	EP	< LOQ	0.41–1.42	0.02–0.04	< LOQ	0.05–0.12	0.14–2.53	0.20–0.57	0.06–0.10	0.03–0.06	< LOQ–0.09	0.07–0.40	0.01	0.49–1.14	This study
			Gut	2.05–16.98	2.74–5.15	0.02–0.03	< LOQ	0.10–0.18	1.22–11.60	99.81–384.32	9.48–15.53	3.76–13.44	0.06–0.21	0.42–1.53	0.07–0.20	0.53–1.19	

*n.d.* not determined

In contrast, Hg gut levels were much higher than those found in the liver in other studies (Table 6). Pb showed variable values among the different studies available, while Zn was consistently lower in our study. Cr and Ni showed similar values to those found by Vitek et al. (2007). Arsenic was studied in rainbow trout (Varol et al., 2017; Robinson et al., 1995), showing a similar concentration to that in our study, while in carp (Jiang et al., 2014), much higher levels were detected. The comparison of literature data with those found in this study clearly showed that metals’ accumulation is fish dependent and site-dependent. However, it is uncommon to find high levels of As and Hg in fish from unpolluted areas, such as in this study. We can tentatively explain this situation considering the geomorphology of the studied site, which showed a derivation from a volcanic eruption that could have led to the release of many metals such as arsenic and mercury (Ma et al., 2019; Witt et al., 2008).

PCA analysis highlighted a different relation between fats and metals’ accumulation. Total lipids in the gut were the most connected with Cd, Hg, and Pb accumulation, with SAFA fraction the closest among fatty acids. Likewise, EP total lipid loadings were influenced by Cd and Pb, with MUFA showing a sufficient correlation with Hg and Pb amounts. A significant number of papers studied the effect of heavy metals on the amount of protein and lipids in fish muscles; only a few data were reported on the correlation between fatty acid of total lipid content and metals accumulation. Khoshnoud et al. (2011) reported a study on two fish species of the Persian Gulf, recording a negative correlation with PUFA% for Pb (− 0.507). Moreover, Rajeshkumar and Li (2018) reported the correlation between metals’ accumulation and the tissue not differentiating among lipids. They found a high correlation among Cd, Pb, and internal organs such as the liver.

The THQ values calculated for individual metals showed no harmful values for human health, considering a conventional portion of approximately 0.036 kg/day EP for a person weighing 67 kg. Thus, the calculated daily intake was below that of the respective reference dose, and these metal levels would not cause any deleterious effect during an entire lifetime. However, some people eat the entire fish (EP + gut), discarding only the head (with the gills) and the backbone; therefore, we calculated the THQ values for the entire fish (EP + gut). In this case, the values for As and Hg largely exceeded 1 (Table 5). Severe contamination of the gut draws attention to possible health risks and should be avoided. The EP’s total THQ was below 1 (0.49), showing no harmful human health values considering all metals and metalloids in the fish.

Arsenic, cadmium, and lead have been classified by the International Agency for Research on Cancer (2012) as both carcinogenic and non-carcinogenic. Considering whole fish consumption, the TR values for As were more significant than 1 × 10^–5^ (Table 5), suggesting a significant cancer risk due to ingestion of As could exist. Although the carcinogenic effects of As exposure are not yet clear, it has been proposed that the As-mediated intracellular biosynthesis of reactive oxygen species, such as free radicals, may be involved in the carcinogenic process induced via DNA damage. Cancer risk was also possible through Cd exposure since its TR values ranged from 10^−5^ in both cases (Table 5). Finally, no risk related to Pb ingestion seemed possible since its TR value was 4.06 × 10^−6^ in the gut and 9.13 × 10^−7^ in the EP.

## Conclusions

The present investigation confirmed that trout bioaccumulate metals at different rates in the gut and muscles. Moreover, the data obtained confirmed that freshwater, even if far from industrial and anthropogenic activities, can lead to the accumulation of heavy metals related to the site's geochemical morphology.

Trout fish were collected from an area originating from a volcanic eruption, followed by siliciclastic depositions in the water basin and complex geomorphology, leading to high levels of Al, As, Cu, Ba, Fe, Hg, and Mn in the riverbed.

However, THQ values were below the limit, and non-carcinogenic risk to humans was not associated with the consumption of the EP of brown trout from the selected freshwater site. When fish are consumed with the gut, adverse effects cannot be excluded due to the high levels of As and Hg accumulated in the gut.

The estimated target cancer risk calculated for As, Cd, and Pb showed no risk if only the EP was consumed, but attention should be given to the levels of As and Cd when consuming the entire fish.

If high, harmful metal concentrations are detected, the amounts of fish consumed should be reconsidered.

## Data Availability

All data generated or analyzed during this study are included in this published article (and its supplementary information files). Moreover, the raw datasets generated during and/or analysed during the current study are available from the corresponding author on reasonable request.
